# Multimodal Protein Constructs for Herbivore Insect Control

**DOI:** 10.3390/toxins4060455

**Published:** 2012-06-12

**Authors:** Frank Sainsbury, Meriem Benchabane, Marie-Claire Goulet, Dominique Michaud

**Affiliations:** Département de phytologie, Centre de recherche en horticulture, Pavillon des Services, Université Laval, Québec QC, G1V 0A6, Canada; Email: frank.sainsbury.1@ulaval.ca (F.B.); mbenchabane@gmail.com (M.B.); marie-claire.goulet@fsaa.ulaval.ca (M.-C.G.)

**Keywords:** insect-resistant transgenic plants, Bt plants, Cry toxins, defense proteins, gene stacking, polyproteins, protein pyramiding, fusion proteins

## Abstract

Transgenic plants expressing combinations of microbial or plant pesticidal proteins represent a promising tool for the efficient, durable control of herbivorous insects. In this review we describe current strategies devised for the heterologous co-expression of pesticidal proteins *in planta*, some of which have already shown usefulness in plant protection. Emphasis is placed on protein engineering strategies involving the insertion of single DNA constructs within the host plant genome. Multimodal fusion proteins integrating complementary pesticidal functions along a unique polypeptide are first considered, taking into account the structural constraints associated with protein or protein domain grafting to biologically active proteins. Strategies that allow for the co- or post-translational release of two or more pesticidal proteins are then considered, including polyprotein precursors releasing free proteins upon proteolytic cleavage, and multicistronic transcripts for the parallel translation of single protein-encoding mRNA sequences.

## 1. Introduction

Large-scale adoption of plant varieties expressing insecticidal Cry toxins from the soil bacterium *Bacillus thuringiensis* (Bt) illustrates the growing importance of insect-resistant transgenic plants in agricultural systems worldwide [1]. Cry toxin-expressing plants (or Bt plants) have been grown in more than 25 countries, on a total area of more than 50 million ha annually [1,2]. Their high degree of resistance to economically important insects, along with the adoption of deployment strategies to delay the onset of genetic resistance among target pest populations, have largely contributed to the success and sustained efficacy of these plants since their first introduction on the market in the mid-1990s [3,4]. After more than 15 years of commercial use, transgenic Bt plant lines still show highly toxic effects against target insects, and documented cases of genetic resistance remain scarce considering the extent of plantations [4].

Resistance monitoring in the field, however, remains challenging [5,6] and genetic adaptation is a threat to the long-term efficacy of any insecticide. The marked lethal effects of Cry toxins exert a strong selection pressure on target pest populations, and genetic resistance to these biopesticides is readily induced among laboratory colonies (e.g., [7,8,9]). Cases of resistance to field-grown Bt plants have also been reported recently in China, India, South Africa and the U.S. [4,10,11,12,13,14,15] despite deployment schemes to prevent resistance, such as the use of Bt lines with high levels of toxin grown along with non-Bt plant refuges for susceptible insects [3]. A reliable strategy to promote long-term effectiveness of transgenic Bt plant lines is to consider these plants as components of much broader, integrated pest management systems involving different approaches for insect control [16,17,18]. A complementary—and conceptually similar—strategy consists of expressing two or more pesticidal proteins in the plants in such a way as to implement an integrated pesticidal system [19,20]. Transgenic plants expressing combinations of Cry toxins interacting with different receptors in the insect midgut were shown to delay the onset of genetic resistance compared to single toxin-plant varieties used alone, sequentially or in mosaics [21,22,23]. Insect adaptation to plant varieties expressing more than one Cry toxin will always remain an issue [3,24,25], but recent surveys for resistance to field-grown Bt plant lines expressing two or three Cry toxins confirm a very low joint resistance allele frequency for the toxins among target populations [26].

The stacking (or “pyramiding”) of Cry toxins *in planta* may also provide improved protection against insects partially susceptible to single toxins [27,28], or help broadening pesticidal effects against different insects to minimize secondary pest infestations upon primary pest control [29,30,31]. From a larger perspective, the pesticidal effects of Cry toxins can be extended by the co-expression of complementary resistance factors with different modes of action [16,32]. For instance, Cry toxins have been expressed in combination with the Bt vegetative insecticidal protein Vip3A [33,34,35], or with plant proteins involved in defense responses to biotic stress agents [36,37,38,39,40]. Pyramiding approaches exclusive of Cry toxins have also been devised, most notably involving alternative Bt endotoxins [41] and plant defense proteins with complementary or synergistic effects such as lectins and protease inhibitors [42,43,44,45,46]. Different strategies have been proposed to co-express several recombinant proteins in plants. Here we review these strategies, with particular emphasis on genetic and protein engineering approaches enabling the coordinated expression of multiple pesticidal proteins under the control of single promoters.

## 2. Transgene Stacking and Pesticidal Protein Pyramiding in Plants

Several studies have reported the successful *in planta* expression of two or three recombinant proteins for pest resistance in plants by “gene stacking” strategies involving sexual crosses between transgenic parental lines bearing distinct transgenes [22,34,38,47,48]. Others have described DNA cloning strategies, transgene cassettes and transformation procedures enabling the co-integration of different resistance transgenes in a single transformation cycle [36,43,45,46,49,50]. Rapid progress has been achieved over the last decade towards the development of multi-transgenic plant lines [51,52,53], but most transgene stacking approaches still present important practical constraints. The most notable are the considerable amount of work and time required for the stable introgression of multiple transgene sequences in plant hybrids, and the onset of gene silencing following the insertion of homologous promoters or multiple T-DNA sequences in recipient genomes [51,54]. Furthermore, the production and identification of multi-transgenic lines that express sufficient and comparable amounts of the different recombinant proteins is not easy, especially given the likelihood of insertional mutagenesis events and position effects that can take place and influence transgene expression in the modified host plants [55].

A possible way to overcome these limitations is to use fusion proteins or polyprotein constructs for the co-expression of distinct proteins under the control of a single promoter. These approaches based on single transgenes present several practical advantages, including the single-step production of transgenic lines with pyramided recombinant traits, the avoidance of multiple T-DNA and promoter sequence insertions, and, most importantly, coordinated expression of different recombinant proteins *in planta*. Three general strategies are currently considered for the co-expression of multiple protein traits encoded by a single transgene sequence in plants: (1) the expression of non-cleavable fusion proteins for the accumulation of multimodal proteins conferring two or several new functional traits; (2) the expression of polyprotein precursors with intrinsic cleavage sites for a co- or post-translational separation of the protein components; and (3) the translation of individual proteins from polycistronic mRNA transcripts.

### 2.1. Multimodal Fusion Proteins

Fusion proteins are composed of at least two covalently attached proteins, protein domains or polypeptides harboring the biological functions of the single components [56]. An interesting example in nature is potato multicystatin, a wound-inducible Cys protease inhibitor expressed in leaves following insect herbivory [57]. This protein is composed of eight protease inhibitory—or cystatin—domains linked within a single polypeptide chain by short ‘linker peptide’ sequences [58]. The eight domains, which presumably were the result of multiple gene duplications in closely related *Solanum* species [59], include hypervariable, rapidly evolving amino acid sites giving the protein a broad range of inhibitory specifities towards insect and plant Cys proteases [60,61]. As shown by *in vitro* inhibitory assays with the model Cys protease papain, the overall stoechiometric ratio of this natural fusion is comparable to the overall ratio of the eight domains taken separately [62], thereby providing a relevant example of the potential of defense or pesticidal proteins as structural modules for multimodal fusion protein design. In the laboratory, recombinant fusion proteins may be engineered by simply ligating the DNA-encoding sequences of two (or more) polypeptides, protein domains or whole proteins, either directly or separated by a linker peptide.

#### 2.1.1. Protein Fusions for Insect Control

Several protein fusions or hybrids have been devised to control herbivorous insects, including Bt toxins or plant defense proteins as primary fusion partners [29,30,31,37,63,64,65,66,67,68,69,70,71,72,73,74,75,76,77,78,79,80,81,82,83,84,85,86,87] (Table 1). For instance, a fusion protein integrating the Bt toxins Cry1B and Cry1Ab was engineered to broaden the insecticidal spectrum of Bt toxin-expressing lines derived from tropical maize varieties [30]. Likewise, toxic peptides and enzymes, such as the spider venom neurotoxin HWTX-I or the *Beauvaria bassiana* protease CDEP2, were fused to Cry1Ac to enhance its pesticidal effects against lepidopteran insects [72,73]. A number of authors have also devised Cry toxin hybrids by protein domain swapping to integrate structural elements of different native toxins within a single chimeric protein sequence. SN19, a fusion protein consisting of specific domains of the Cry1Ba and Cry1Ia Bt toxins, was designed based on this approach [66] and used to produce transgenic potato lines resistant to both coleopteran and lepidopteran pests [31]. Another Cry toxin hybrid active against insects of different orders was developed recently based on Cry1Ab modified with part of the Cry3A variable region [64].

**Table 1:** Hybrid and fusion proteins devised for herbivorous insect control—Selected examples from the current literature

Fusion partners	Intended uses / Improved effects	Refs.
*Hybrid proteins*		
Cry1Ab toxin modified with domain III of Cry1C	Improved efficacy against *Spodoptera exigua*	[63]
Cry1Ab toxin modified with C-terminal region of Cry1Ac	Improved efficacy and range against Lepidoptera	[29]
Cry1Ab toxin modified with part of Cry3A variable region	Resistance to insects of different orders	[64]
Cry1Ac or Cry1E modified with domain III of Cry1C	Improved efficacy against *Spodoptera exigua*	[65]
Cry1Ba toxin modified with domain II of Cry1Ia	Resistance to insects of different orders	[31,66]
Cry1Ca, Cry1Fb and Cry1Ba modified with Cry1Ac domain III	Improved efficacy against *Heliothis virescens*	[67]
Cry1Ea toxin modified with part of Cry1Ca toxin domain III	Improved efficacy against *Spodoptera litura*	[68]
Cry hybrid SN19 modified with domain II of Cry1Ba	Resistance to insects of different orders	[69]
Sunflower multicystatin integrating gourd trypsin inhibitor	Broader inhibition of Lepidoptera midgut proteases	[70]
*Bi- or multimodal translational fusions*		
Cry1B and Cry1Ab toxins	Durability and broader range against Lepidoptera	[30]
Cry1Ac toxin and galactose-binding domain of ricin B chain	Improved efficacy and broader insecticidal range	[71]
Cry1Ac toxin and cowpea trypsin inhibitor CpTI	Dual effect against cabbage worms and durability	[37]
Cry1Ac toxin and spider venom neurotoxin HWTX-I	Dual effect against *Plutella xylostella*	[72]
Cry1Ac toxin and *Beauveria bassiana* subtilisin CDEP2	Dual effect against *Helicoverpa armigera*	[73]
Cry1Ac toxin and baculoviral polyhedrin	Increased stability and expression in *E. coli*	[74]
Cry1Ab toxin C-ter peptide and spider toxin ACTX-Ar1	Improved efficacy and range against Lepidoptera	[75,76,77,78]
Snowdrop lectin and arthropod peptide toxins	Delivery of toxic peptides to the haemolymph	[79,80,81,82,83]
Soybean cystatin N2 and GSII lectin	Dual effect against *Callosobruchus maculatus*	[84]
Various plant and animal cysteine protease inhibitors	Broader inhibition of Thysanoptera midgut proteases	[85]
Tomato cathepsin D inhibitor and corn cystatin II	Broader inhibition of Coleoptera midgut proteases	[86]
Oryzacystatin I and potato carboxypeptidase inhibitor domains	Broader inhibition of Coleoptera midgut proteases	[87]

Pesticidal effects for fusion proteins employing plant proteins or protein domains have also been reported. For instance, Mehlo *et al.* [71] fused a galactose-binding domain from ricin B to Cry1Ac to improve its binding properties in the insect midgut epithelium and to broaden its pesticidal effects against different lepidopteran insects. Translational fusions with different plant lectins and/or protease inhibitors have been devised as a way to improve the biological effects of the individual components [70,84,86,87] or to stabilize protein partners prone to degradation along the plant–insect continuum [84,85,88]. Plant lectins have also been used as fusion partners to deliver toxic polypeptides, such as spider or scorpion venom neurotoxins, into the haemolymph of various insects [79,80,81,82,83]. These promising developments, along with rapid advances in protein structural biology and molecular modeling allowing for the design of potentially effective fusions *in silico* [89], confirm the potential of fusion protein design as a realistic avenue for the production of recombinant proteins integrating multiple pesticidal functions.

#### 2.1.2. Non-Cleavable Linker Peptides

On the other hand, protein domain grafting may alter the structure and function of some proteins, with possible unexpected effects on the resulting protein fusions. For instance, the *Allium sativum* lectin “ASAL” showed an altered glycosylation pattern, decreased thermal stability and no bacteriocidal activity when fused to the small ubiquitin related modifier (SUMO) peptide [90]. Similarly, the inhibitory activity of rice cystatin I against papain and herbivorous pest Cys proteases was substantially lowered when the 39-amino acids inhibitor of carboxypeptidase A, potato carboxypeptidase inhibitor, was grafted at the *C*-terminus [87]. A strategy to avoid such effects consists of adding a linker peptide to seperate the protein partners in such a way as to avoid detrimental non-specific physicochemical interactions between the non-cognate polypeptides [91,92]. Computational approaches have been developed to devise linker peptides, such as the program LINKER, which generates synthetic linker sequences from known protein structures based on user-defined requirements [93,94]. Another approach takes into account the important issue of proteolytic lability by a systematic screening of candidate linker sequences against the substrate specificities of proteases listed in the MEROPS peptidase database [95,96].

Empirical assessments with different linker peptides will possibly remain the most robust approach to identify linkers adapted to specific macromolecular contexts, but current knowledge on the properties of natural and synthetic linkers already provides helpful hints for successful outputs [92,97]. Gly-rich peptide motifs, such as the well-characterized pentapeptide motif Gly–Gly–Gly–Gly–Ser, are known to ensure a certain flexibility of protein fusions and are useful in preventing functional disturbance and destabilization of the domain partners [98]. By comparison, Pro-rich motifs confer rigidity to the proteins and are useful in maintaining a minimal distance and preventing potential non-specific interactions between the fusion partners [91,92]. Data are still scarce about the performance of linker peptides in plant biotechnology, but a number of studies suggest the potential of Gly-containing linkers to produce protease-resistant fusions with dual pesticidal activities against insects or pathogens [84,85,86,99,100]. A rigid linker of fungal origin has also been used to produce a bifunctional protein, resulting in a dual inhibitor of herbivorous pest proteases that was partially resistant to proteolytic processing [101].

Despite promising results, fusion proteins integrating linker peptides might not always be the optimal alternative to linker-free, tail-to-tail translational fusions. The choice and number of amino acids used to devise an effective linker are critical because this structural element may itself impact the overall performance of the fusion partners [102,103], fusion protein yield [104], tendency to aggregate [103], or robustness of the fusion product in terms of thermal stability [95,104] and resistance to proteolysis [95,103,105]. Biological context-dependent effects, including the host organism used for expression, further add to the complexity of linker sequence design and stress the need for more empirical data on natural and synthetic linkers. For example, a helical linker that allows for more activity of fusion partners than a flexible linker was also more sensitive to proteolysis in yeast [103] and prone to autocatalytic cleavage under certain pH conditions [105]. A trade-off might be unavoidable, in some cases, between the possible gains obtained with linker peptides in terms of yield and activity of the fusion partners, and different constraints impacting their structural stability in the host environment selected for expression.

### 2.2. Polyprotein Precursors

**Figure 1:**
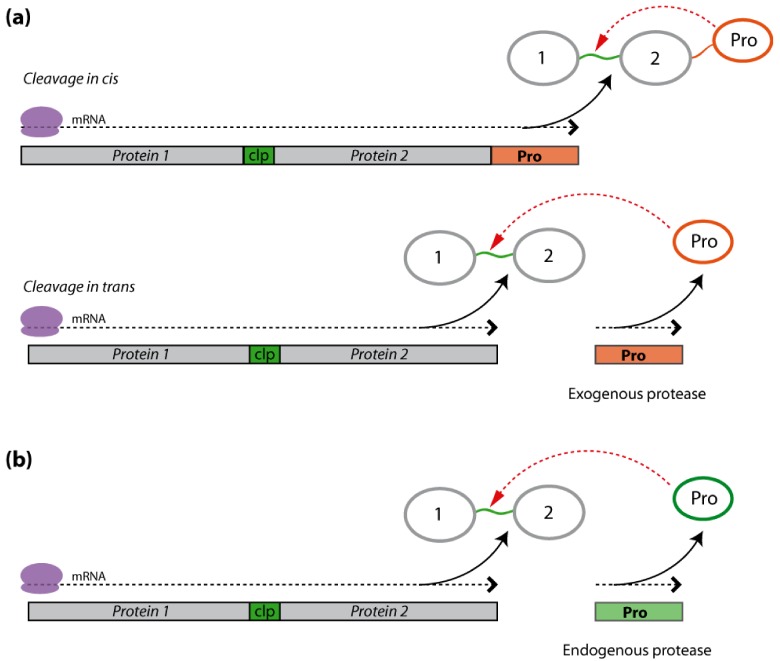
Cleavable polyprotein precursor constructs for the heterologous co-expression of two hypothetical pesticidal proteins, Protein 1 and Protein 2, in transgenic plants. The polyprotein precursor includes a cleavable linker peptide (clp) (in green) between the two protein moieties, which is post-translationally processed by exogenous or endogenous proteases (Pro) to release the two mature proteins. (**a**) Exogenous protease-mediated cleavage. The polyprotein precursor may be cleaved off by a recombinant protease expressed as part of a processing functional unit (cleavage *in cis*) or after the integration of an independent, co-expressed protease-encoding transgene (cleavage *in trans*). (**b**) Endogenous protease-mediated cleavage. Alternatively, the mature proteins may be released by cleavage of a clp recognized by the host plant endogenous proteases. Black arrows on panels (**a**) and (**b**) indicate the direction of ribosome-mediated mRNA translation. Red arrows point to protease-susceptible sites on cleavable linker peptides.

One possible way to avoid such trade-offs is to express polyprotein precursors with cleavable linkers between the proteins of interest. This approach is based on the gene/protein expression strategy adopted by some viruses to ensure a quantitative balance of protein components *in vivo* [106]. The protein expression pattern of these viruses first involves the synthesis of a large polypeptide precursor, which is then processed to release the mature proteins [107,108]. Recombinant polyproteins may be engineered to include the coding sequences of multiple proteins separated by accessory DNA sequences encoding a proteolysis-susceptible peptide for processing by either host endogenous proteases or an exogenously supplied recombinant protease (Figure 1).

#### 2.2.1. Exogenous Protease Cleavage

An exogenous protease with high specificity towards linker cleavage sites may be supplied as a self-processing component of the same polyprotein as the proteins of interest for cleavage *in cis*, or from a co-expressed DNA sequence as a viral accessory endoprotease for cleavage *in trans* (Figure 1a). Examples of viral proteases used in plant biotechnology are the well-characterized tobacco etch virus nuclear inclusion NIa protease [109] and the cowpea mosaic virus 24 K proteinase [110]. These proteases excise themselves from viral polyproteins during natural infections, where they then go on to process the remaining polyprotein fragments to release the individual proteins [111,112]. For instance, the NIa protease specifically recognizes and cleaves the heptapeptidic sequence “Glu–X–X–Tyr–X–Gln–↓[Gly or Ser]”, with the scissile bond located between the Gln–Gly (or Gln-Ser) dipeptide motif [113,114]. In practice, the NIa protease may be co-expressed *in planta* along with an engineered polyprotein bearing the corresponding protease-cleavable motifs between the *C*- and *N*-termini of the proteins to be produced. NIa protease/polyprotein precursors have been expressed in several plant systems, notably allowing for the ectopic implementation of short metabolic pathways in transgenic plant cells [115], the coordinate expression of interacting transcription factors to activate endogenous metabolic pathways [116], and the differential subcellular translocation of recombinant proteins harboring distinct targeting signals after polyprotein processing [117]. NIa/polyproteins have also been used to co-express defense proteins in plants, such as the coat proteins of different plant viruses [118,119] and a number of antimicrobial peptides and proteins [120,121].

#### 2.2.2. Endogenous Protease Cleavage

An alternative to supplying exogenous protease functions consists of integrating a cleavable, plant protease-sensitive linker between the proteins to express and targeting the resulting polyprotein precursor to the endoplasmic reticulum for processing by endogenous proteases along the cell secretory pathway [122] (Figure 1b). The antidigestive protease inhibitors cowpea trypsin inhibitor and rice cystatin I have been produced in Arabidopsis using this approach by expressing a single translational fusion product for the two proteins separated by a cleavable peptide from the plant metallothionein-like protein, PsMTa [101]. In a similar way, a polyprotein precursor including the antimicrobial defensins DmAMP1 and RsAFP2 linked by a seed protein cleavable sequence from *Impatiens balsamina* was shown to drive the accumulation of free and active forms of the two defensins in the extracellular milieu of transgenic Arabidopsis leaves [123,124,125]. These findings, along with a recently described approach involving ubiquitin-derived linker sequences for processing by endogenous deubiquitinating proteases [126], underline the potential of cleavable polyproteins sensitive to host endogenous proteases for the coordinate expression and release of multiple recombinant proteins, including pesticidal proteins, in plants.

### 2.3. Polycistronic Constructs

**Figure 2:**
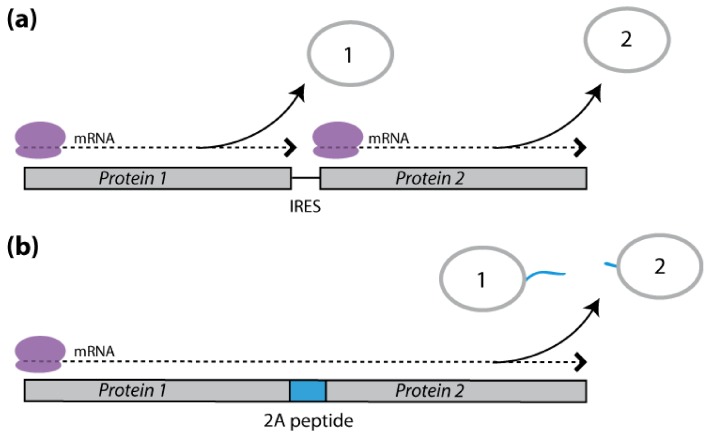
Polycistronic constructs for the heterologous co-expression of two hypothetical pesticidal proteins, Protein 1 and Protein 2, in transgenic plants. (**a**) IRES-mediated translation. An internal ribosome entry site (IRES) sequence is included between the codingsequences of Protein 1 and Protein 2 to drive a cap-independent, internal initiation of Protein 2 translation, in parallel to Protein 1 cap-dependent translation initiated at the polycistron transcript 5' end. (**b**) 2A peptide-mediated translation. A viral 2A peptide sequence is included between the coding sequences of Protein 1 and Protein 2 to induce ‘ribosomal skipping’ during translation leading to the co-translational release of the two proteins.

Polycistronic DNA constructs for the expression of different proteins from a single mRNA transcript also show potential to generate transgenic plant lines producing multiple recombinant proteins [51]. Unlike polyprotein-encoding constructs, polycistronic constructs drive the accumulation of free recombinant proteins in host tissues, with no need for an endogenous or recombinant accessory protease acting co- or post-translationally on the translated polypeptide product. Two main approaches have been described to express recombinant proteins in plants using polycistronic mRNAs, both of which make use of viral protein expression strategies (Figure 2). The first approach involves a viral structural RNA motif, the so-called internal ribosome entry site (IRES) [127]. The second approach involves the 2A catalytic peptide of foot-and-mouth disease virus and related picornaviruses [128].

#### 2.3.1. IRES-Mediated mRNA Translation

IRES motifs direct ribosomes to initiate translation in a cap-independent manner, at internal positions within polycistronic transcripts [129] (Figure 2a). A number of IRES motifs from plant [130,131] and animal [132] viruses have successfully been used to direct the expression of multiple recombinant proteins in plants and plant cells. Bicistronic constructs have been used, for instance, to engineer nematode pest resistance in tobacco by the co-expression of cowpea trypsin inhibitor and rice cystatin I [133], or to facilitate the implementation of abiotic stress tolerance in cell cultures and transgenic plants by linking the detection of a marker gene with the expression of a stress-related protein [134,135]. However, IRES-based approaches present practical limitations associated with the highly variable efficacy of IRES motifs *in planta*, their taxonomic origin and the plant tissue selected for protein expression [131,133,134,136,137]. Perhaps the most prominent limitation preventing the widespread use of IRES motifs in plant biotechnology is their relative inefficiency. Internally initiated translation is generally low compared to cap-dependent translation [133] and is, therefore, hardly compatible with plant protection strategies requiring sufficient and comparable amounts of different pesticidal proteins.

#### 2.3.2. 2A Peptide-Mediated mRNA Translation

A straightforward way to ensure a balanced translation of polycistron-encoded proteins may be to use the 20-amino acids 2A peptide of foot-and-mouth disease virus [51,138]. This peptide mediates a “ribosomal skip” during viral transcript translation that results in a co-translational, non-proteolytic dissociation of the encoded proteins [139]. This unique biochemical process prevents a peptide bond to form between the last two amino acids of the viral peptide and causes the nascent polypeptide to dissociate from the ribosomal translational complex while allowing mRNA translation to continue [139,140]. Polycistronic protein constructs involving the 2A peptide may be engineered *in vitro* by insertion of this peptide between the recombinant proteins to be expressed (Figure 2b). The correct processing of 2A peptides in plants was initially reported for transgenic tobacco lines expressing combinations of the model reporter proteins β-glucuronidase, chloramphenicol acetyltransferase and green fluorescent protein [141,142]. Several studies then reported the successful co-expression of useful proteins by this approach, for applications as diverse as the heterologous expression of vaccines and antibodies [143,144,145], the engineering of carotenoid biosynthetic pathways [137,146], storage protein enrichment to increase the nutritional value of plant foods [147], and the expression of stress or pesticidal proteins conferring abiotic stress tolerance [148] or pest resistance [125,149]. The presence of several non-cognate amino acids at the *C*-terminus of the upstream protein(s) following mRNA translation still represents a practical limitation to the 2A peptide technology [128], but strategies have been devised in recent years to remove this extension, relying on the addition of cleavage sites between the *C*-terminus and the viral peptide [150,151]. A number of factors should make the 2A peptide expression strategy particularly useful for the implementation of multiple protein resistance traits in plants [138,152]. The most obvious are the possibility of integrating several recombinant proteins and 2A peptide connections within the same construct [153,154], and the relatively efficient self-processing activity of 2A peptide-containing polyproteins in plant systems [142]. Other factors are the greater efficiency of this expression system compared to the IRES motif strategy [137], and the possible targeting of recombinant proteins to different subcellular locations after their co-translational release from the ribosomal complex [153,154,155].

## 3. Concluding Remarks

Numerous studies have illustrated the potential of multimodal protein constructs for the implementation of multiple defense protein traits in plants. Expression strategies based on stable fusion proteins or cleavable polyprotein precursors have been described, as well as alternative strategies involving polycistronic DNA constructs for the co-translational release of functionally distinct proteins. Accumulating data in forthcoming years should further demonstrate the potential of these approaches for a durable control of plant pests and pathogens. One forthcoming issue will be to implement the resulting control tools in realistic conditions, not only in terms of efficiency and applicability, but also taking into account the possible impacts of multifunctional protein systems at the ecosystem level [156]. Plant protection strategies involving pesticidal proteins such as the Cry toxins have the obvious advantage of being highly specific to target pests. Hybrid protein constructs integrating multiple, and sometimes less specific, recombinant traits could by contrast exert undue effects on non-target organisms. A further challenge in the field will be to find the right balance between the efficacy, durability and specificity requirements of each pest control problem.
